# A Machine Learning-Based Approach for Classification of Focal Splenic Lesions Based on Their CT Features

**DOI:** 10.3389/fvets.2022.872618

**Published:** 2022-05-02

**Authors:** Silvia Burti, Alessandro Zotti, Federico Bonsembiante, Barbara Contiero, Tommaso Banzato

**Affiliations:** ^1^Department of Animal Medicine, Production and Health, University of Padua, Viale dell'Università 16, Padua, Italy; ^2^Department of Comparative Biomedicine and Food Science, University of Padua, Viale dell'Università 16, Padua, Italy

**Keywords:** spleen, computed tomography, focal lesion, sarcoma, decision tree, factorial discriminant analysis

## Abstract

The aim of the study was to describe the CT features of focal splenic lesions (FSLs) in dogs in order to predict lesion histotype. Dogs that underwent a CT scan and had a FSL diagnosis by cytology or histopathology were retrospectively included in the study. For the statistical analysis the cases were divided into four groups, based on the results of cytopatholoy or hystopathology, namely: nodular hyperplasia (NH), other benign lesions (OBLs), sarcoma (SA), round cell tumour (RCT). Several qualitative and quantitative CT features were described for each case. The relationship occurring between each individual CT feature and the histopathological groups was explred by means of c chi-square test for the count data and by means of Kruskal-Wallis or ANOVA for the continuous data. Furthermore, the main features of each group were described using factorial discriminant analysis, and a decision tree for lesion classification was then developed. Sarcomas were characterised by large dimensions, a cystic appearance and an overall low post contrast-enhancement. NH and OBLs were characterised by small dimensions, a solid appearance and a high post-contrast enhancement. OBLs showed higher post-contrast values than NH. Lastly, RCTs did not exhibit any distinctive CT features. The proposed decision tree had a high accuracy for the classification of SA (0.89) and a moderate accuracy for the classification of OBLs and NH (0.79), whereas it was unable to classify RCTs. The results of the factorial analysis and the proposed decision tree could help the clinician in classifying FSLs based on their CT features. A definitive FSL diagnosis can only be obtained by microscopic examination of the spleen.

## Introduction

Focal splenic lesions (FSLs) are common in dogs, especially in elderly subjects. Most FSLs (51%) are benign; the most common histotypes are haematoma, nodular hyperplasia, and myelolipoma (1–3). Haemangiosarcoma is reported as the most common primary malignant tumour of the spleen, accounting for almost 80% of malignant FSLs (1, 2), followed by fibrosarcoma and leiomyosarcoma. Splenic metastases (from other primary sarcomas, carcinomas or neuroendocrine tumours in most cases) are less common, accounting for 1–6% of the total of the FSLs (3).

Despite FSLs being a common finding in canine ultrasound (US) and computed tomography (CT) (4) there is a general paucity of studies systematically describing their imaging features. No specific US features are reported as useful in distinguishing between different FSL histotypes (5). Previous studies describing the CT features of FSLs have reported conflicting results. Fife et al. (6), reported that, in dual-phase CT imaging, a FSL with a post-contrast Hounsfield Unit value lower than 55 is most likely malignant. However, Jones et al. (7), reported no dual-phase CT features as useful in the distinction between benign and malignant lesions. Kutara et al. (2), using triple-phase CT imaging, reported some CT features (lesion volume and homogeneous contrast enhancement) as useful in differentiating between haematoma, nodular hyperplasia, haemangiosarcoma and undifferentiated sarcoma in dogs. Lastly, Lee et al. (8) reported triple-phase CT, combined with ultrasonography, as useful in the differentiation between benign and malignant lesions.

In the last few years, an increasing number of research papers exploring the possible applications of machine learning in veterinary radiology have been published (9–15). Research in this field has mostly been focused on the automatic classification of radiographic images (14, 16, 17), the distinction between benign and malignant brain lesions on MRI (10, 18), and the classification of liver focal lesion types on CT images (19). To the best of the authors' knowledge, the approach of applying machine learning to classify splenic lesions based on their CT appearance has not yet been explored.

In such a scenario, the aims of this study are: (1) to describe the CT features of FSLs in dogs; (2) to use machine learning algorithms to describe the complex relationship existing between different FSL histotypes and their CT features; and (3) to develop an easy-to-use algorithm for classifying FSLs based on their CT features.

## Materials and Methods

### Study Population

The medical records of 62 dogs (32 males and 30 females – mean age 10.4 ± 2.3 years) referred to the Pedrani Veterinary Clinic (Via Caldierino 14, Zugliano, Vicenza, Italy) and to the Veterinary Teaching Hospital of the University of Padua (Viale Dell'Università 16, Legnaro, Padua, Italy) between June 2015 and November 2021 were prospectively collected. Criteria for inclusion in the study were: (1) a CT scan was conducted, (2) cytopathological and/or histopathological diagnosis of the splenic lesion. Exclusion criteria were: (1) chemotherapy at the time of the CT scan; (2) non-diagnostic cytopathological samples or equivocal cytopathological diagnosis. Patient signalment was recorded for each animal. The dogs belonged to several different breeds (31 mixed breeds, four Labrador Retrievers, three Golden Retrievers, two Boxers, two Bernese Mountain dogs, two German Shepherds, two Cockers, two Cane Corso, and one each of Fox Terrier, Yorkshire Terrier, English setter, Whippet, Great Dane, Weimaraner, Pointer, Jack Russell Terrier, Belgian Shepherd Dog, Australian Shepherd Dog, Shih Tzu, Lakeland Terrier and Hovawart). Six dogs were excluded because they were receiving chemotherapy at the time of the CT scan, and four were excluded because the cytopathological samples resulted as non-diagnostic. Of the remaining 52 dogs, 16 had a final diagnosis of nodular hyperplasia, six of normal splenic parenchyma, five of extramedullary haematopoiesis, three of haematoma, two of lymphoma, two of histiocytic sarcoma, two of mastocytoma, one of mesenchymal neoplasia, one of plasma-cell neoplasia, and 14 of sarcoma (five sarcoma, four stromal sarcoma, three hemangiosarcoma, one leiomyosarcoma, and one myxoid liposarcoma). The cases were grouped into four broader histological categories for the statistical analysis: nodular hyperplasia NH, 16 cases; other benign lesions (OBLs), 14 cases; round cell tumour (RCT), eight cases, sarcoma (SA), 14 cases.

All the methods were carried out in compliance with the relevant guidelines and regulations. This study was conducted respecting the Italian Legislative Decree N° 26/2014 (transposing EU Directive 2010/63/EU). Nevertheless, since the data used in this study were part of routine clinical activity, no ethical committee approval was required. Informed consent for personal data processing was obtained from the owners.

### Cytopathological and Histopathological Examination

Thirty-three splenic masses were sampled through ultrasound-guided fine needle aspiration for cytological assessment. Twenty one-gauge needles were always used. Cytological slides were obtained by smearing the aspirates on glass slides, which were subsequently air-dried, stained with May-Grünwald-Giemsa stain and cover-slipped. All the cytological slides were evaluated by the same cytologist (FB). Cytology was always performed immediately after the CT scan. Histopathology was not performed in any of these cases.

Twenty-one splenic masses were sampled through ultrasound-guided Tru-cut biopsy for histological assessment. Formalin-fixed tissue samples were dehydrated in a graded ethanol series and embedded in paraffin. Four-μm-thick sections were stained with haematoxylin and eosin and evaluated by one pathologist.

### Computed Tomography Examination

Three different scanners were used to perform the CT examinations: Asteion super 4 (Toshiba Medical System Corporation), at the Veterinary Teaching Hospital; Revolution ACT, General Electric Medical System), and Optima CT 520 Series (General Electric Medical System) at the Pedrani Veterinary Clinic. The scanning protocols were slightly different for the different scanners. The protocols for the Asteion super 4 were: helical acquisition mode, exposure time of 0.725 s, voltage of 120 kV, amperage of 150 mA, and slice thickness of 1–3 mm. For the Revolution ACT, were: exposure time of 0.725 s, voltage of 100 kV, amperage of 100 mA, and slice thickness of 1–2.5 mm. Lastly for the Optima CT 520 Series were: exposure time of 0.725 s, voltage of 120 kV, amperage of 180 mA, and slice thickness of 1–3 mm.

All the dogs underwent a 12-h fasting period prior to examination. All the examinations were performed on anaesthetised subjects placed in ventral recumbency. Contrast medium (Ioversol 350 mg/ml, Optiray 350, Liebel-Flarsheim Company LLC, USA) was administered at the dosage of 660 mg/kg through two different modalities depending on the facility: (1) via an injector at the Pedrani Veterinary Clinic; (2) manually injected intravenously as a bolus at the Veterinary Teaching Hospital. Pre-contrast and delayed phase CT scans, the latter starting 30–50 s after the end of the contrast medium injection, were always performed.

All the images were stored as digital imaging and communication in medicine (DICOM) files.

### Image Analysis

All the scans were retrieved using a picture archiving and communication system (PACS). All the images were reviewed in a soft tissue window (WW: 400 HU - WL: 40HU) using a commercially available software (Horos v3.3.6). In the case of multiple lesions in the same dog, the largest sampled lesion was described.

The following qualitative features were evaluated: (1) margins (well- or ill-defined); (2) surface (regular or irregular); (3) appearance (solid or cyst-like) - the lesion was classified as “cyst-like” in the presence of at least one area with a measured Hounsfield Unit (HU) value similar to that of the animal's gallbladder (representing possible necrosis or haemorrhage) (20); (4) splenic lymph-nodes appearance (normal or abnormal) – splenic lymph nodes were classified as abnormal if any of the following changes were evident: a) lymphadenomegaly (the dimensions of the splenic lymph nodes were subjectively compared to the surrounding abdominal lymph nodes), b) heterogeneous appearance c) round or irregular shape; (5) homogeneity of contrast-medium distribution inside the lesion (homogeneous or heterogeneous); (6) enhancement pattern (prevalently central, rim enhancement, or diffuse distribution).

The following quantitative characteristics were evaluated: (1) attenuation (measured as an HU value) of the tomographically normal splenic parenchyma, in both the pre-contrast and the delayed phase; (2) attenuation (mean HU value) of the lesion in both the pre-contrast and the delayed phase; (3) maximum transverse diameter; (4) volume - the shape of the lesion was considered to be an ellipsoid and the formula $V\;=\;\frac{4}{3}\pi \;\left(height/2^{*}width/2^{*}length/2\right)$ was applied (21); (5) attenuation of the lesion compared to that of the radiologically normal splenic parenchyma in the pre-contrast images (hypoattenuating, isoattenuating or hyperattenuating); (6) enhancement degree of the lesion compared to that of the radiologically normal splenic parenchyma in post-contrast images (hypoenhancing, isoenhancing or hyperenhancing). The attenuation and the enhancement degree of the lesion were determined based on the difference between the mean HU value measured on the lesion and the HU value measured on the radiologically normal splenic parenchyma. The lesions were classified as: a) isoattenuating/isoenhancing if the difference fell in the ±10 HU range; b) hyperattenuating/hyperenhancing with a difference greater than +10 HU; c) hypoattenuating/hypoenhancing if the difference was lower than −10 HU (7, 22). The HU values were measured in three circular regions of interest (ROIs), in both the normal and in the pathological parenchyma, carefully avoiding cystic regions and vascular structures. The same ROIs were selected in pre- and post-contrast images. The size of the ROI was manually adjusted for each case.

The CT features were evaluated separately by two of the authors of this study (SB: with 4 years' experience in diagnostic imaging and AZ, with 20 years' experience in diagnostic imaging). The reviewers were blinded to the results of the histopathological examination.

### Statistical Analysis

To compare the differences between the four diagnostic categories, the count data expressed as percentages were analysed with a chi-square test (or Fisher's exact test when there were fewer than 5 units of data). The quantitative variables were assessed for normality using Shapiro-Wilks test. Differences between the four diagnostic categories were analysed with a one-way analysis of variance (ANOVA) for normally distributed data, whereas the non-parametric Kruskal-Wallis test was used for non-normally distributed data. A Bonferroni *post-hoc* pairwise comparison test was performed. A *p* < *0.05* was considered as statistically significant. The analyses were conducted with SAS 9.4 (SAS Institute Inc., Cary, NC, USA).

To describe the complex relationship existing between all the different CT features and the histopathological groups, two different supervised machine learning techniques were applied. The first to be used was a dimensionality reduction technique, known as factorial discriminant analysis (FDA). This technique was chosen in order to identify which of the CT features best discriminated between the four histopathological categories. FDA aims to identify different linear combinations of original features (components - F) that provide the best possible separation of two or more classes of units. A coefficient is assigned to each original variable based on its relative ability to discriminate between different groups. Different components are computed and, usually, the first two components explain most of the variance in the dataset. The correlations between the original variables and components were calculated in our study and coefficient values of |r|>0.6 and >0.5 for the first component and the second component, respectively were considered significant. Classification of all the cases based on the first two components is plotted on a Cartesian plane, where the position on the x-axis is determined by the results of F1 and the position on the y-axis is determined by the results of F2, and this enables the discrimination ability of the analysis to be visually assessed. Lastly, the centroids (i.e., the arithmetic mean positions of all the points in a group) are plotted. The further away each centroid is from the 0 of the Cartesian axes and from the centroids of the other groups, the better is the discrimination ability of the analysis is for that group. The factorial discriminant analysis was performed using XLStat (Addinsoft 2022, XLSTAT statistical and data analysis solution, New York, USA).

Decision tree analysis was then performed to detect the best discriminating CT features (a recursive partitioning method was adopted using the rpart package of R – https://cran.r-project.org/web/packages/rpart/vignettes/longintro.pdf, and a three-step procedure was applied to build the decision tree: (1) the features that provided the best data splitting were selected; (2)10-fold cross-validation was used to prune the decision tree having the lowest number of branches and the lowest misclassification rate (19); (3) a confusion matrix was built by comparing the values of actual vs predicted samples (obtained from the decision tree classification), and some quality indices regarding model performance were calculated (sensitivity, specificity, accuracy and misclassification rate).

## Results

**Figure 1:**
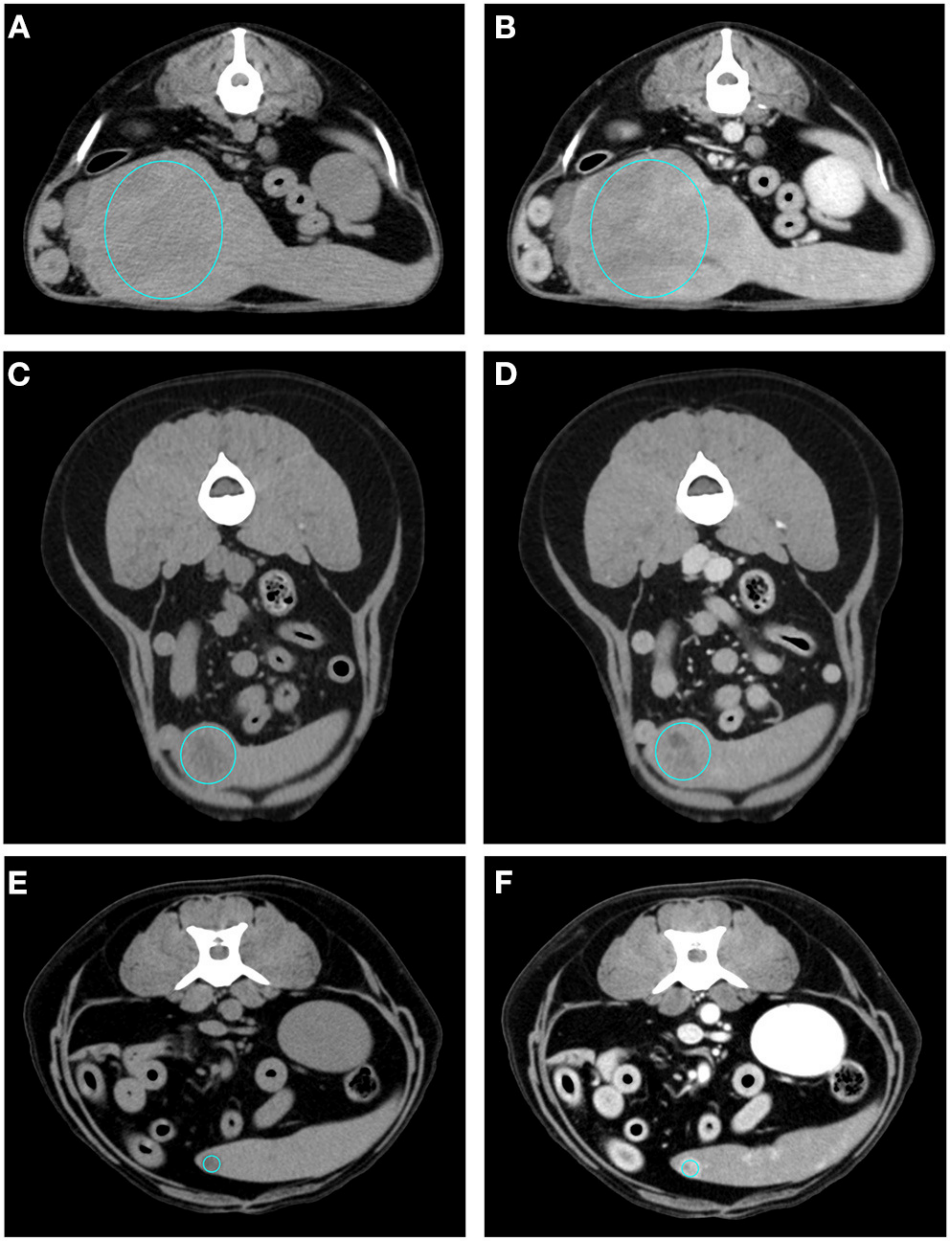
Pre- **(A)** and post- **(B)** contrast images of NH showing isoattenuation and hypoenhancement, diffuse enhancement pattern with heterogeneous distribution, well-defined margins, irregular surface, and a cyst-like appearance. Pre- **(C)** and post- **(D)** contrast images of NH showing hypoattenuation and hypoenhancement, diffuse enhancement pattern, with heterogeneous distribution, ill-defined margins, regular surface, and cyst-like appearance. Pre- **(E)** and post- **(F)** contrast images of NH showing hypoattenuation and hyperenhancement, rim enhancement pattern with heterogeneous distribution, well-defined margins, irregular surface, and solid appearance. The ROI is placed inside the lesions.

**Figure 2:**
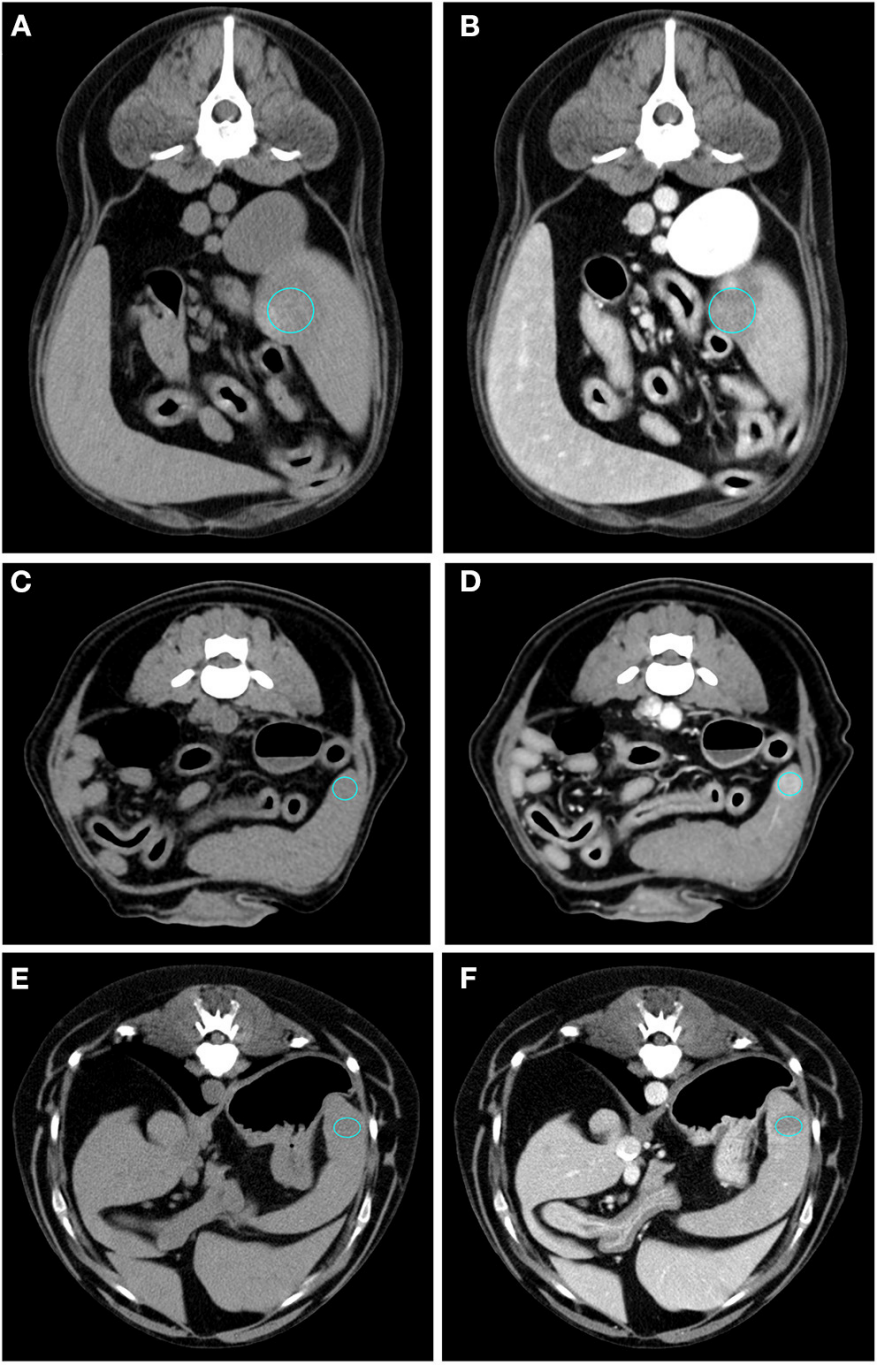
Pre- **(A)** and post- **(B)** contrast images of an OBL (diagnosed as extramedullary haematopoiesis) showing hypoattenuation and hypoenhancement, diffuse enhancement pattern with heterogeneous distribution, well-defined margins, irregular surface, and solid appearance. Pre- **(C)** and post- **(D)** contrast images of an OBL (diagnosed as extramedullary haematopoiesis) showing isoattenuation and hyperenhancement, diffuse enhancement pattern with homogeneous distribution, well-defined margins, regular surface, and solid appearance. Pre- **(E)** and post- **(F)** contrast images of an OBL (diagnosed as haematoma) showing hypoattenuation and hypoenhancement, diffuse enhancement pattern with heterogeneous distribution, well-defined margins, regular surface, and cyst-like appearance. The ROI is placed inside the lesions.

**Figure 3:**
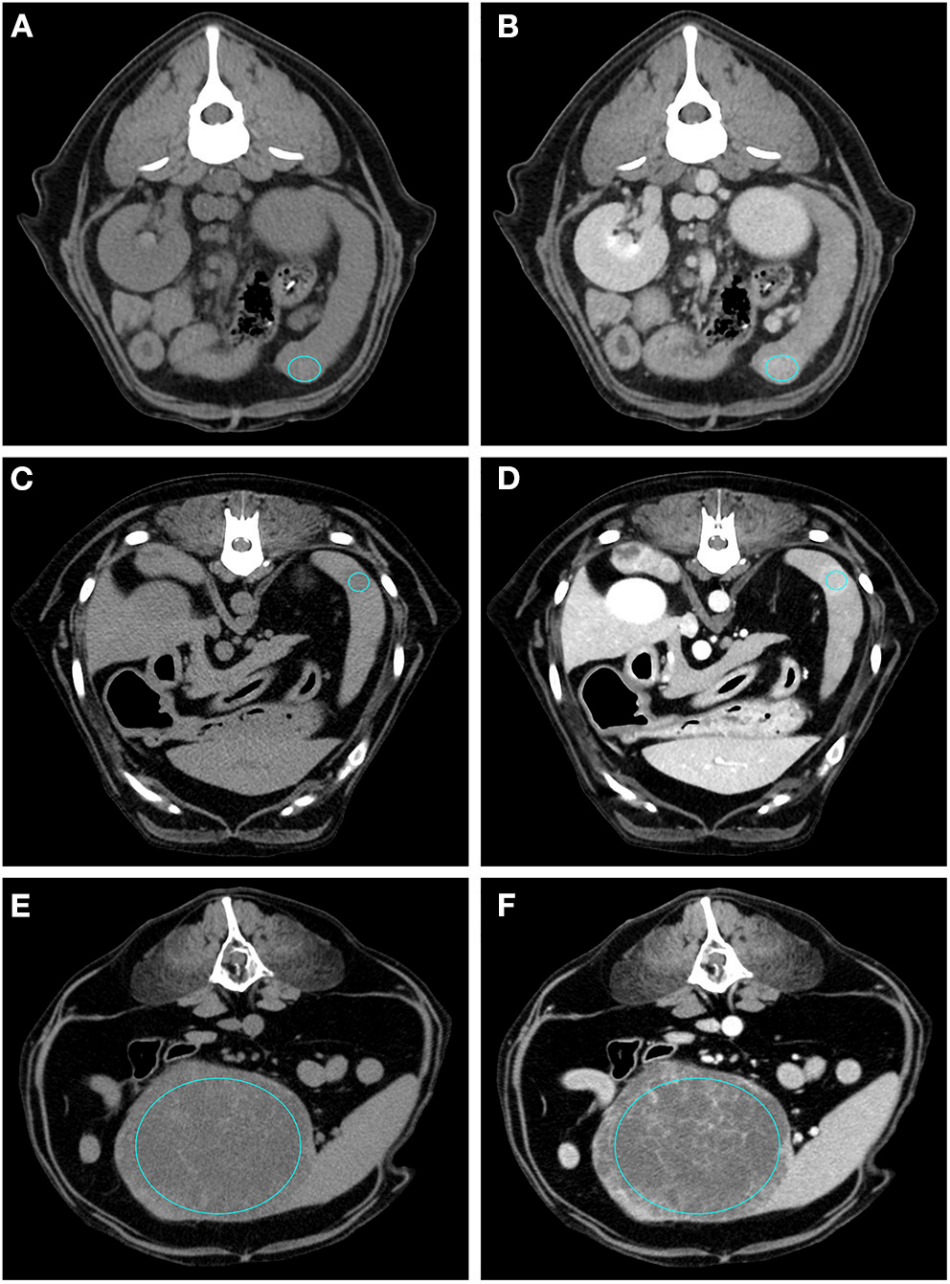
Pre- **(A)** and post- **(B)** contrast images of a RCT (diagnosed as lymphoma) showing isoattenuation and hyperenhancement, diffuse enhancement pattern with homogeneous distribution, ill-defined margins, irregular surface, and solid appearance. Pre- **(C)** and post- **(D)** contrast images of a RCT (diagnosed as mastocytoma) showing hypoattenuation and hyperenhancement, rim enhancement pattern, with heterogeneous distribution, well-defined margins, regular surface, and solid appearance. Pre- **(E)** and post- **(F)** contrast images of a RCT (diagnosed as mesenchymal neoplasia) showing hypoattenuation and hypoenhancement, diffuse enhancement pattern with heterogeneous distribution, well-defined margins, irregular surface, and cyst-like appearance. The ROI is placed inside the lesions.

**Figure 4:**
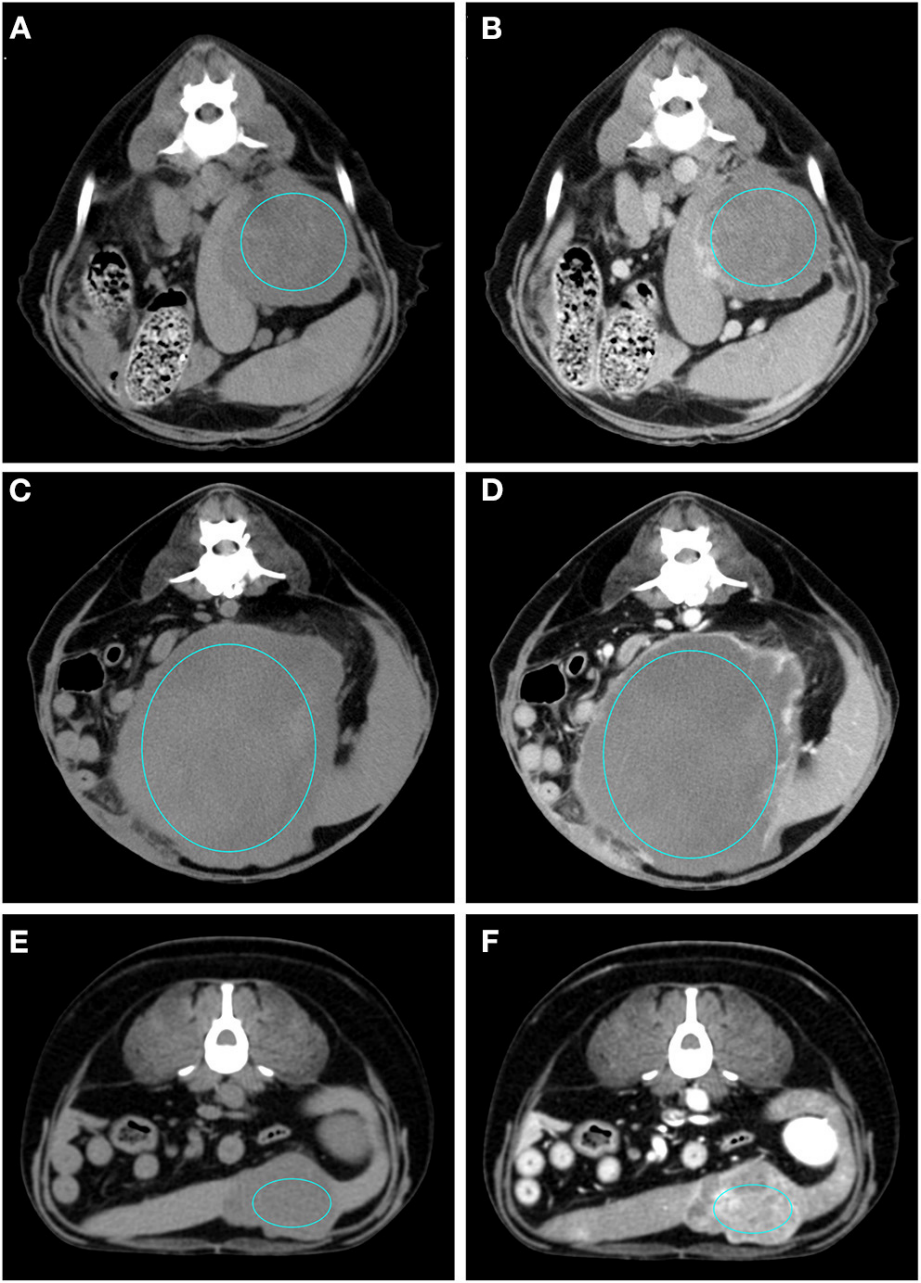
Pre- **(A)** and post- **(B)** contrast images of a sarcoma (diagnosed as haemangiosarcoma) showing hypoattenuation and hypoenhancement, rim enhancement pattern with heterogeneous distribution, well-defined margins, irregular surface, and cyst-like appearance. Pre- **(C)** and post- **(D)** contrast images of a sarcoma (diagnosed as sarcoma) showing isoattenuation and hypoenhancement, diffuse enhancement pattern with heterogeneous distribution, well-defined margins, irregular surface, and cyst-like appearance. Pre- **(E)** and post- **(F)** contrast images of a sarcoma (diagnosed as stromal sarcoma) showing hypoattenuation and hyperenhancement, diffuse enhancement pattern with heterogeneous distribution, well-defined margins, irregular surface, and cyst-like appearance. The ROI is placed inside the lesions.

The results of the analysis of the qualitative and quantitative features of the images, along with their *p*-values, are reported in Tables 1, 2, respectively. Pre- and post- contrast example images for each histopathological category are reported in Figures 1–4. Among the qualitative features, only the surface (χ^2^ = 8.71; *p*-value = 0.033) and the appearance (χ^2^ = 12.98; *p*-value = 0.005) showed statistically significant differences between histopathological groups. In particular, the main differences were found between OBLs and SAs for both the surface and the appearance. In fact, almost all (13/14) the SAs had an irregular surface and a cyst-like appearance. Instead, OBLs showed mainly a solid appearance (11/14) whereas surface was almost evenly distributed between regular (8/14) and irregular (6/14). The margins (χ^2^ = 5.12; *p*-value = 0.163), lymph nodes (*p*-value = 0.169), post contrast homogeneity (χ^2^ = 4.37; *p*-value = 0.224), and enhancement pattern (χ^2^ = 1.10; *p*-value = 0.776) did not show statistically significant differences between the histopathological groups.

**Table 1:** Qualitative features, along with cytological or histological classification.

	**Category**					
	**Nodular hyperplasia *(n = 16)***	**Other benign lesions ^†^ *(n = 14)***	**Round cells tumors^+^ *(n = 8)***	**Sarcoma^++^ *(n = 14)***	**Total *(n = 52)***	***p*-value**
Margins^*^						0.163
Well-defined	9 (56%)	10 (71%)	6 (75%)	13 (93%)	38 (73%)	
Ill-defined	7 (44%)	4 (29%)	2 (25%)	1 (7%)	14 (27%)	
Surface^*^						0.033
Regular	7 (44%)	8 (57%)	2 (25%)	1 (7%)	18 (35%)	
Irregular	9 (56%)^ab^	6 (43%)^b^	6 (75%)^ab^	13 (93%)^a^	34 (65%)	
Appearance^*^						0.005
Solid	10 (62%)	11 (79%)	5 (62%)	2 (14%)	28 (54%)	
Cyst-like	6 (38%)^ab^	3 (21%)^b^	3 (38%)^ab^	12 (86%)^a^	24 (46%)	
Lymph-nodes^**^						0.169
Normal	13 (81%)	10 (71%)	5 (63%)	6 (43%)	34 (65%)	
Abnormal	3 (19%)	4 (29%)	3 (38%)	8 (57%)	18 (35%)	
Post-contrast homogeneity^*^						0.224
Homogeneous	5 (31%)	5 (36%)	1 (14%)	1 (7%)	12 (23%)	
Heterogeneous	11 (69%)	9 (64%)	7 (86%)	13 (93%)	40 (77%)	
Enhacement pattern^*^						0.776
Diffuse enhancement	12 (75%)	11 (79%)	5 (63%)	9 (64%)	37 (71%)	
Rim enhancement	4	3	3	5	15 (29%)	
Central enhancement	0	0	0	0	0	
Pre-contrast attenuation^**^						0.171
Hypoattenuating	7 (44%)	6 (43%)	5 (63%)	11 (79%)	29 (56%)	
Isoattenuating	9 (56%)	6 (43%)	3 (38%)	3 (21%)	21 (40%)	
Hyperattenuating	0	2 (14%)	0	0	2 (4%)	
Post-contrast attenuation^**^						0.309
Hypoenhancing	8 (50%)	7 (50%)	5 (63%)	12 (86%)	32 (61%)	
Isoenhancing	1 (6%)	1 (7%)	1 (13%)	0	3 (6%)	
Hyperenhancing	7 (44%)	6 (43%)	2 (25%)	2 (14%)	17 (33%)	

*Different letters along columns mean significant different values for p < 0.05*.

* *k proportion test*.

** *Fisher's exact test*.

† *Other benign lesions = 6 normal parenchyma, 5 extramedullary haematopoiesis, 3 haematomas*.

+ *Round cell tumour = 2 mastocytomas, 2 lymphomas, 2 histiocytic sarcomas, 1 mesenchymal neoplasia, 1 plasma-cell neoplasia*.

++ *Sarcoma = 5 sarcomas, 4 stromal sarcomas, 3 hemangiosarcomas, 1 leiomyosarcoma, 1 myxoid liposarcoma*.

**Table 2:** Quantitative features, along with cytological or histological classification.

	**Category**					
	**Nodular hyperplasia *(n = 16)***	**Other benign lesions^†^ *(n = 14)***	**Round cell tumour^+^ *(n = 8)***	**Sarcoma^++^ *(n = 14)***	**Total *(n = 52)***	***p*-value**
Maximum dimension (cm)^*^	2.17 (1.65–2.97)^b^	5.09 (2.50–8.65)^ab^	5.47 (1.22–12.37)^ab^	10.67 (7.57–16.00)^a^	4.59 (1.96–10.28)	0.001
Ellipsoid volume (cm^3^)^*^	2.77 (1.45–8.32)^b^	23.45 (5.50–315.99)^b^	72.20 (0.62–538.71)^b^	375.24 (152.03–1387.69)^a^	23.45 (2.39–350.86)	0.001
HU value of pre-contrast normal spleen^**^	61.03 ± 6.01	63.11 ± 11.59	55.11 ± 9.34	54.18 ± 7.06	58.83 ± 9.18	0.026
HU value of post-contrast normal spleen^**^	106.29 ± 17.05	108.61 ± 24.31	116.65 ± 20.88	108.46 ± 14.74	109.09 ± 19.05	0.665
HU value of pre-contrast lesion^**^	49.59 ± 12.67^ab^	60.75 ± 24.59^a^	42.49 ± 10.91^ab^	33.26 ± 11.29^b^	47.10 ± 18.95	0.001
HU value of post-contrast lesion^**^	93.90 ± 29.19^ab^	106.77 ± 47.24^a^	88.85 ± 31.28^ab^	60.17 ± 32.23^b^	87.51 ± 39.22	0.010

*Different letters along columns mean significant different values for p < 0.05*.

* *Kruskal-Wallis test*.

** *One-way ANOVA*.

† *Other benign lesions = 6 normal parenchyma, 5 extramedullary haematopoiesis, 3 haematomas*.

+ *Round cell tumour = 2 mastocytomas, 2 lymphomas, 2 histiocytic sarcomas, 1 mesenchymal neoplasia, 1 plasma-cell neoplasia*.

++ *Sarcoma = 5 sarcomas, 4 stromal sarcomas, 3 haemangiosarcomas, 1 leiomyosarcoma, 1 myxoid liposarcoma*.

*HU = Hounsfield Unit*.

**Figure 5:**
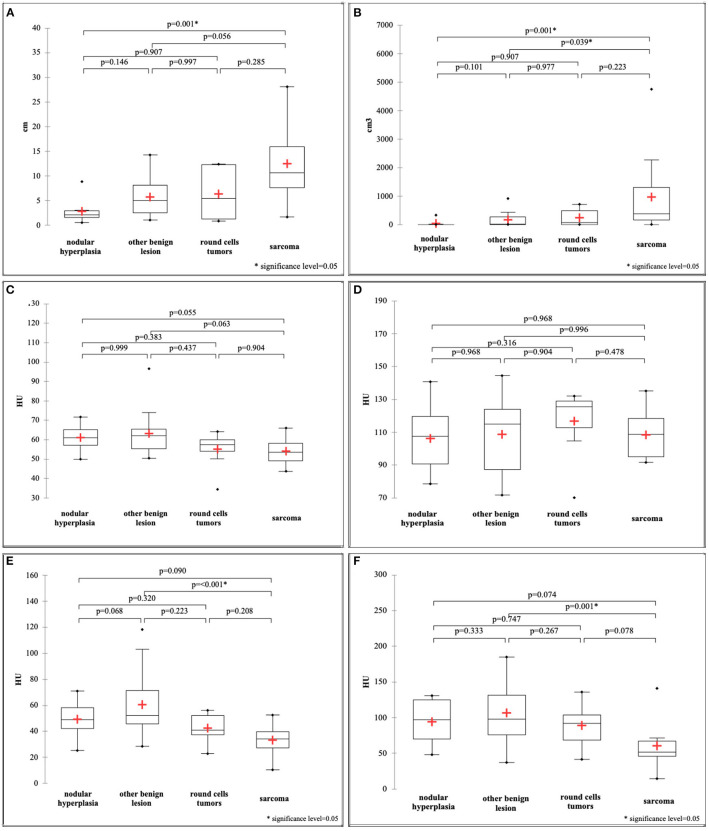
Box and whisker plot of the maximum dimension **(A)**, ellipsoid volume **(B)**, HU value of the pre-contrast normal spleen **(C)**, HU value of the post-contrast normal spleen **(D)**, HU value of the pre-contrast lesion **(E)**, HU value of the post-contrast lesion **(F)**.

Maximum dimension and ellipsoid volume showed a non-normal distribution and, therefore, differences between the groups were calculated with the Kruskal-Wallis test. All the remaining variables showed a normal distribution and, therefore, differences were evaluated with the ANOVA. Most of the quantitative features revealed significant differences between the groups: HU value of pre-contrast normal spleen (*F* = 3.37; *p*-value = 0.026), HU value of pre-contrast lesion (*F* = 6.97; *p*-value = 0.001), HU value of post-contrast lesion (*F* = 4.20; *p*-value = 0.01), maximum dimension (*k* = 16.13; *p*-value = 0.001), and ellipsoid volume (*k* = 16.94; *p*-value = 0.001). Only the HU value of the post-contrast normal spleen showed no statistically significant differences between groups (*F* = 0.53; *p*-value = 0.665). Box-plots of all the quantitative variables are reported in Figure 5. It seems clear from analysis of the box plots that differences are mainly evident between sarcomas and other lesions. In particular the only statistically significant differences in the HU values of pre-contrast lesions and of post-contrast lesions are between OBLs and SAs. Only differences between NH and SAs were evident for both maximum dimension and ellipsoid volume (two highly correlated values).

**Figure 6:**
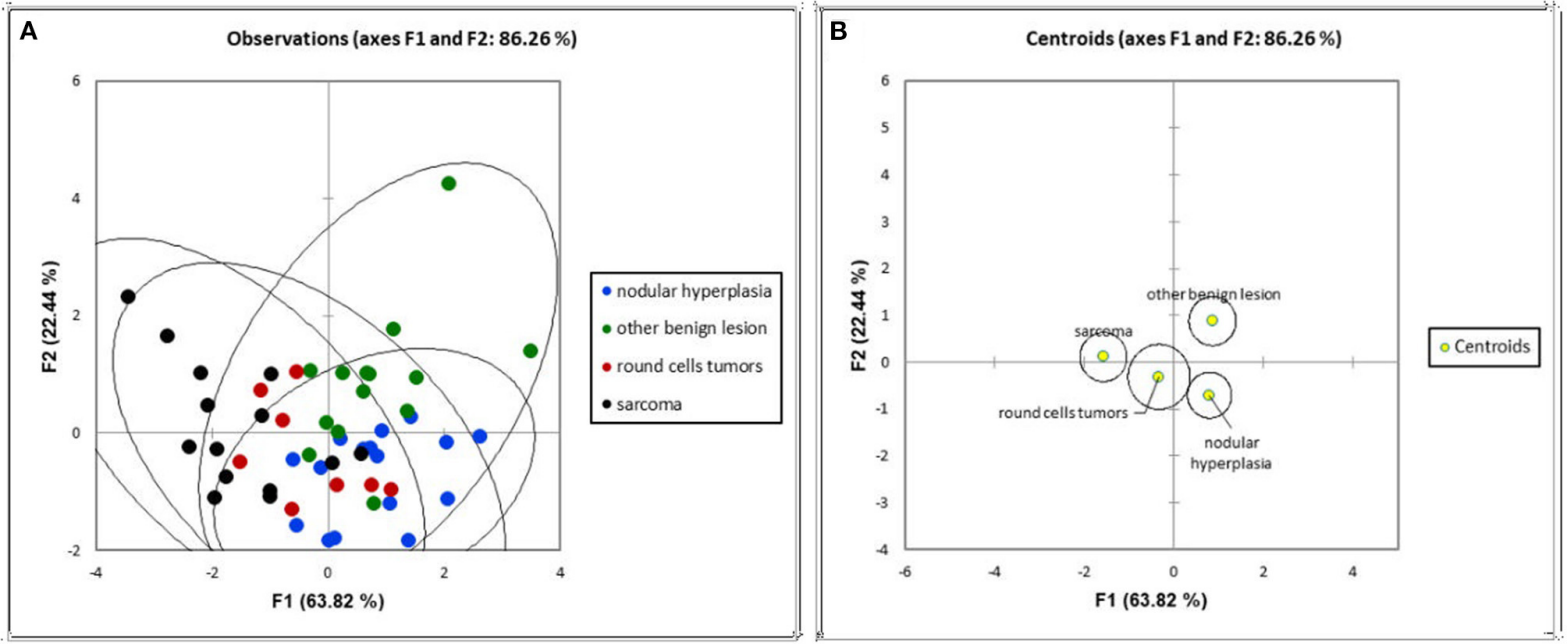
Distribution of the cases **(A)** and of the centroids **(B)** based on the F1 and F2 components of the factorial discriminant analysis classification.

The first two main components of the FDA (called F1 and F2) together explained about 86% of the total variability. The coefficients for F1 and F2 are reported in Table 3. The first component, explaining 63.82% of the total variability, is positively correlated (|*r*|>0.6) mainly with the HU value of the pre- and post-contrast lesion and with a solid appearance of the lesion, and is inversely correlated with maximum dimension and cystic appearance. The second component, explaining only 22% of the total variability, is moderately related only to pre-contrast hyperattenuatuation (|*r*|>0.5). Case distribution using the Cartesian system, based on classification by the two main components, is represented in Figure 6. From the graph in Figure 6A, and from the positions of the centroids (Figure 6B), it appears evident that the sarcoma group lies (almost completely) in the negative part of the x-axis, and is therefore associated with characteristics such as larger maximum dimensions and a cyst like appearance (that had a negative correlation to F1). The NH and OBL groups are both positioned in the positive part of the x-axis, and are therefore mainly characterised by smaller dimensions, higher pre- and post-contrast mean HU values and a solid appearance (positive correlation with F1). Furthermore, OBLs and NH are separated on the y-axis (F2), with OBLs exhibiting higher values than nodular hyperplasia. Lastly, RCTs are located in the centre of the Cartesian axis system and thus do not show any distinctive CT feature. Lastly, although an overall tendency for each group is noted, the large superimposition of the cases around the 0 on the Cartesian axes indicates that the subdivision of SA, OBL, and NH based on the CT features is suboptimal.

**Table 3:** F1 and F2 values of the factorial discriminant analysis based on 8 qualitative and 5 quantitative predictors.

	**F1**	**F2**
Well-defined margins	−0.384	0.255
Ill-defined margins	0.384	−0.255
Regular surface	0.54	0.189
Irregular surface	−0.54	−0.189
Solid appearance	0.636	0.144
Cyst-like apperance	−0.636	−0.144
Normal lymph nodes	0.413	−0.161
Abnormal lymph nodes	−0.413	0.161
Homogeneous distribution	0.381	0.089
Heterogeneous distribution	−0.381	−0.089
Diffuse contrast-enhancement pattern	0.174	0.075
Rim enhancement pattern	−0.174	−0.075
Hypoattenuation	−0.421	−0.006
Isoattenuation	0.338	−0.202
Hyperattenuation	0.225	0.528
Hypoenhancement	−0.426	0.028
Isoenhancement	0.143	−0.035
Hyperenhancement	0.371	−0.011
Maximum dimension	−0.769	0.38
HU value of pre-contrast normal spleen	0.533	0.191
HU value of post-contrast normal spleen	−0.064	0.018
HU value of pre-contrast lesion	0.69	0.401
HU value of post-contrast lesion	0.593	0.178

**Figure 7:**
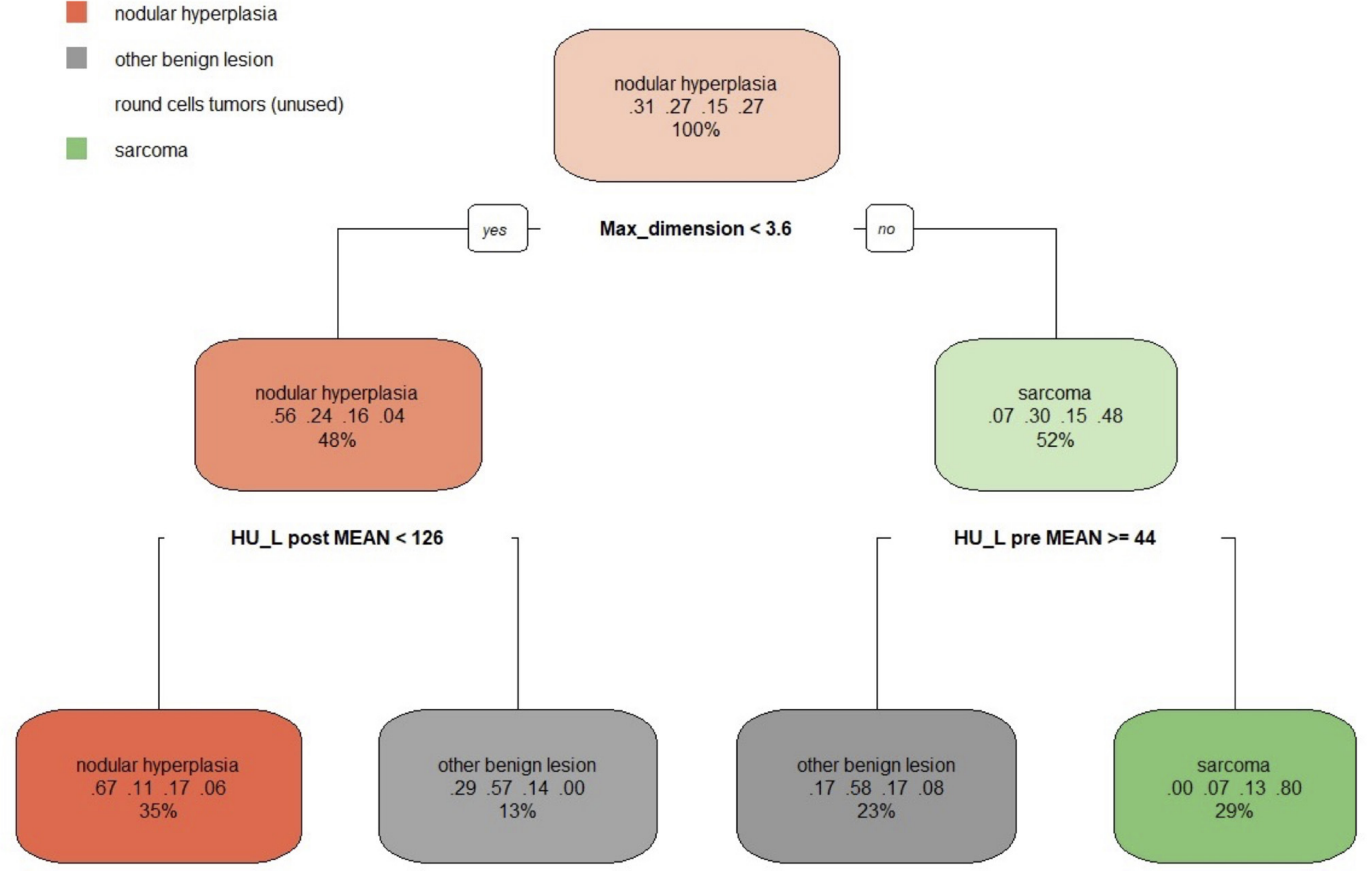
The machine learning-based decision tree developed on the qualitative and the quantitative CT features of the focal splenic lesions. The second line in each box shows the probability of each class at that node (i.e., the probability of the class conditioned on the node) and the third line shows the percentage of observations used at that node.

The decision tree resulting from the analysis is reported in Figure 7. Three variables (max dimension, mean HU value of the pre-contrast lesion and HU value of the post-contrast lesion) were used for classification. Following the first split (max. dimensions <3.6 cm), the decision tree classified 48% of the cases as nodular hyperplasia (max. dimension <3.6 cm) and 52% of the cases as sarcoma (max dimension > 3.6 cm). Of these 48% classified as NH 56% were actually NH, 24% were OBLs, 16% were RCTs and only 4% were SAs. Instead, of the 52% of the cases classified as SA 7% were NH, 30% were OBLs, 15% were RCTs, and 48% were actually SAs. Following the second split on the left (mean HU value of the post-contrast lesion <126), 35% of the cases were classified as NH (67% actually NH, 11% actually OBL, 17% actually RCT, 6% actually SA) and 13% (HU lesion post mean > 126) were classified as OBL (29% actually NH, 57% actually OBL, 14% actually RCT, and 0% actually SA). On another decision tree branch (mean HU values of pre-contrast lesion ≥44), 23% of the cases were classified as OBLs (17% actually NH, 58% actually OBL, 17% actually RCT, and 8% actually SA). On another secondary branch the algorithm classified 29% of the cases as sarcomas (0% actually NH, 7% actually OBL, 13% RCT, 80% actually SA).

Therefore, the following observations summarise the main findings of the decision tree. If the lesion is smaller than 3.6 cm and has a post-contrast HU value lower than 126 there, is a 67% chance that is NH (a 78% combined chance that it is benign if classing NH and OBLs together). If the lesion is smaller than 3.6 cm and has a post-contrast HU value higher than 126, there is a 57% chance that it is an OBL (an 86% combined chance that it is benign if classing OBLs and NH together). Instead, if the lesion is larger than 3.6 cm and has a mean pre-contrast mean HU value higher or equal to 44, there is a 58% chance the lesion is an OBL and a cumulative 75% chance it is benign. Lastly, if the lesion is larger than 3.6 cm and has a pre-contrast HU lower than 44, there is an 80% chance it is a SA and a cumulative 93% chance it is malignant (classing RCT and SA together). Not surprisingly, the algorithm did not identify any specific feature enabling the differentiation of RCTs from the remaining histopathological categories. The overall accuracy of the decision tree, when reapplied on the original data, was 0.67 and the *k* was 0.54. The sensitivity, the specificity and the balanced accuracy of the decision tree for each FSL category is reported in Tables 4, 5.

**Table 4:** Confusion matrix that summarises the performance of the machine learning-based decision tree, giving the number of predicted cases.

		**Actual**				
		**Nodular hyperplasia**	**Other benign lesions**	**Round cells tumors**	**Sarcoma**	**Total**
**Predicted**	Nodular hyperplasia	12	2	3	1	18
	Other benign lesions	4	11	3	1	19
	Round cells tumour	0	0	0	0	0
	Sarcoma	0	1	2	12	15
	**Total**	16	14	8	14	52

**Table 5:** Results of the classification of the focal splenic lesions based on the machine learning-based decision tree.

	**Nodular hyperplasia *(n = 16)***	**Other benign lesions *(n = 14)***	**Round cells tumors *(n = 8)***	**Sarcoma *(n = 14)***
Sensitivity	0.75	0.79	0.00	0.86
Specificity	0.83	0.79	1.00	0.92
Balanced accuracy	0.79	0.79	0.50	0.89

## Discussion

The complex relationship occurring between the CT features and the FSL histotypes were described using both a classical statistical and a machine learning-based approach. The classical statistical analysis revealed some significant differences between groups, mainly for the quantitative features, whereas no significant differences, with the exception of surface and appearance, were evident for the qualitative features. The machine learning algorithms substantially confirmed the results of the classical statistical analysis; indeed, all the features included in both in the FDA and the decision tree resulted as significantly different between the different groups in the classical statistical analysis tests. Nonetheless, the main advantage of FDA is that it allows identification of subtler trends than the classical statistical analysis does. As a result of the FDA, it emerged that while SAs were characterized both by larger dimensions and a cyst-like appearance, strong similarities were evident in the appearance of NH, OBLs and SAs than based on classical statistical analysis. Furthermore, the decision tree provided a straightforward and easy-to-use chart that could be directly used to classify lesions based on their CT features with a very high accuracy for SAs and a moderate accuracy for OBLs and NH, while RCTs could not be classified through the decision tree.

NH nodules are reported as having a variable appearance (both homogeneous and heterogeneous) and as being markedly hyperenhancing on post-contrast CT images (2, 6, 7). The results of both the discriminant analysis and the decision tree confirm such findings and, in fact, NHs were characterised by higher pre- and post-contrast HU values and a smaller volume than SAs were. Interestingly, but not unexpectedly since several extramedullary haematopoiesis cases were present in the OBL group, NH showed an overall lower enhancement than the OBLs did.

In this paper we placed all benign lesions other than NH (i.e. haematomas and extramedullary haematopoiesis) in the OBL group and, therefore, a straightforward comparison with the features reported by other authors for this lesion category is not possible. Haematomas are reported as mainly heterogeneously enhancing masses in all phases by both Kutara et al. (2) and Jones et al. (7). Splenic extramedullary haematopoiesis nodules have been described as having a very variable appearance and as hyperenhancing in all phases (4). The CT features of all the other possible OBLs (e.g., splenitis, lipoma, etc.) have not yet been reported in the literature.

The literature reporting the CT features of malignant splenic lesions is fragmentary and different authors have grouped malignant lesions differently. Both Fife et al. (6) and Jones et al. (7), grouped all malignant lesions into a single category during statistical analysis. To date only Kutara et al. (2) have considered sarcoma and haemangiosarcoma as individual categories during statistical analysis. In the present study, all the sarcoma cases were grouped together due to the presence of only three haemangiosarcomas in the database. To the best of the authors' knowledge, this is the first manuscript considering RCT as an individual category for the analysis. The results of the present study confirm the finding that sarcomas have a lower attenuation and larger dimensions compared to benign splenic lesions (both NH and OBLs). Instead, RCTs did not show any distinctive CT feature. Therefore, splenic lesions should not be classified based on their CT features alone since other diagnostic procedures (e.g., cytology) are necessary to determine the histotypes. Interestingly, the splenic lymph nodes were normal in 63 % of RCTs and 43% of SAs, whereas the lymph nodes were abnormal in 23% of the benign lesions. Therefore, it is the author's opinion that lymph node evaluation also has poor value in determining whether a lesion is benign or malignant.

Fife et al. (6), reported the presence of abdominal effusion as significantly correlated with the presence of malignant splenic lesions. Instead, abdominal effusion was not detected in any of the cases included in the present study and, therefore, the significance of such a finding was not evaluated. The significance of abdominal effusion was also not evaluated by Lee et al. (8), and Jones et al. (7).

Contrast-enhanced ultrasound has seldom been reported as useful in the differentiation between benign and malignant FSLs (23, 24). However, these reports are largely outdated and based on a relatively low number of cases (26 and 29 cases, respectively), and the full efficacy of this imaging technique has yet to be proven.

In human medicine, FSL diagnosis based on diagnostic imaging findings alone poses challenges similar to those of the veterinary context (25). Nonetheless, the combination of CT, MRI and 18F-FDG PET/CT enables attainment of a high degree of confidence for lesion characterisation (25). However, due to the limited availability of such imaging devices and of properly developed techniques (18F-FDG PET/CT) in veterinary medicine, the MRI features of FSLs have seldom been described in dogs (26). It is likely that the combination of different imaging techniques could enable attainment of a higher degree of confidence in FSL diagnosis also in the veterinary field. Nonetheless, this will require a broad standardisation of the features of FSLs in each individual diagnostic imaging technique.

Most of the results of the present study are in agreement with those reported in the literature. In particular, Lee et al. (8) reported the HU values of the lesion in pre-contrast phase, along with the regular and irregular margination of the lesion as statistically significant features in the distinction between malignant and benign tumors. Results of this study confirmed, the appearance of the surface and the HU values of the lesion during pre-contrast phase to be useful in the distinction among the four considered pathological categories.

Fife et al. (6) reported the HU values of the lesion during pre- and post-contrast phase as significantly different between malignant lesions, hematomas and NH. Nevertheless, the authors found a threshold of <55HU in post contrast scans for classification of malignant lesions. In our study, the decision tree, used a threshold of <44 HU to distinguish between sarcoma and OBL, and of <126 HU to distinguish between NH and OBL.

Lastly, Kutara et al. (2) found the size of the mass as statistically different between NH, hematomas, hemangiosarcoma, and undifferentiated sarcoma. In particular, the size was smaller for NH. Our results confirmed that the size is significantly smaller in case of NH, with a 3.6 cm cut-off value.

One limitation of the present study is that, since most of the FSLs were occasional findings on CT scans performed for other reasons (e.g., staging of neoplasia), the lesions were evaluated only in the pre-contrast and in the delayed phase and no arterial phase was available for the selected cases. Previous reports on the CT features of FSL (2, 6–8) used both dual-phase (6, 7) and triple-phase (2, 8) scanning protocols. To the best of the authors' knowledge, no CT features specifically related to the arterial phase have yet been shown as useful in differentiating between the different FSL histotypes.

Another limitation is that no specific RCTs were differentiated by the proposed decision tree. A possible explanation of this is that several different histotypes (lymphoma, histiocytic sarcoma, mastocytoma, mesenchymal neoplasia, plasma-cell neoplasia) with different imaging features were included in the RCT category. By including a larger number of cases in a future study, a larger number of groups could likely be considered during analysis, which would therefore provide a more detailed description of the CT features of FSLs.

The third possible limitation is related to the use of cytopathology to classify the cases. Indeed, the agreement between cytopathological and hystopathological diagnoses of the spleen is reported to be only moderate (Cohen's Kappa = 0.473) (27). In the present study histopathology was performed only in 19 cases, while the remainder 33 cases were evaluated only by means of cytopathology. To improve the classification accuracy both non-diagnostic cases and cases with doubtful cytological diagnosis were excluded. It is the authors' opinion that, including only cases with high quality cytopathological samples increase the diagnostic accuracy of the cytological exam.

## Conclusions

The CT features of different groups of FSL have been described and analysed using both classical statistical analysis and machine learning algorithms. SAs are characterised by large dimensions, a cystic appearance and an overall low post-contrast enhancement. NH and OBLs are characterised by small dimensions, a solid appearance and a high post-contrast enhancement. OBLs show higher post- contrast values than NH. Lastly, RCTs do not exhibit any distinctive CT features. A straightforward, easy-to-use decision tree for classifying FSLs is proposed.

## Data Availability Statement

The raw data supporting the conclusions of this article will be made available by the authors, upon reasonable request.

## Ethics Statement

This study was conducted respecting Italian Legislative Decree N° 26/2014 (transposing EU directive 2010/63/EU). Since the data used in this study were part of routine clinical activity, no ethical committee approval was required. Informed consent for personal data processing was obtained from the owners.

## Author Contributions

TB, SB, and AZ conceived the study, performed the CT scans, and drafted and revised the manuscript. FB revised the manuscript and standardized part of the cytological examinations. BC drafted and revised the manuscript and performed the statistical analysis. All the authors contributed to the article and approved the submitted version.

## Funding

The present paper is part of a project funded by a research grant from the Department of Animal Medicine, Production and Health – MAPS, University of Padua, Italy: SID – Zotti 2018 (€ 32,000; Application of deep-learning algorithms in pet animal diagnostic imaging).

## Conflict of Interest

The authors declare that the research was conducted in the absence of any commercial or financial relationships that could be construed as a potential conflict of interest.

## Publisher's Note

All claims expressed in this article are solely those of the authors and do not necessarily represent those of their affiliated organizations, or those of the publisher, the editors and the reviewers. Any product that may be evaluated in this article, or claim that may be made by its manufacturer, is not guaranteed or endorsed by the publisher.
